# Investigating the Responses of Human Epithelial Cells to Predatory Bacteria

**DOI:** 10.1038/srep33485

**Published:** 2016-09-15

**Authors:** Ajay K. Monnappa, Wasimul Bari, Seong Yeol Choi, Robert J. Mitchell

**Affiliations:** 1 Department of Biological Sciences, School of Life Sciences, Ulsan National Institute of Science and Technology, 44919, South Korea

## Abstract

One beguiling alternative to antibiotics for treating multi-drug resistant infections are *Bdellovibrio*-and-like-organisms (BALOs), predatory bacteria known to attack human pathogens. Consequently, in this study, the responses from four cell lines (three human and one mouse) were characterized during an exposure to different predatory bacteria, *Bdellovibrio bacteriovorus* HD100, *Bacteriovorus* BY1 and *Bacteriovorax stolpii* EB1. TNF-α levels were induced in Raw 264.7 mouse macrophage cultures with each predator, but paled in comparison to those obtained with *E. coli*. This was true even though the latter strain was added at an 11.1-fold lower concentration (*p* < 0.01). Likewise, *E. coli* led to a significant (54%) loss in the Raw 264.7 murine macrophage viability while the predatory strains had no impact. Tests with various epithelial cells, including NuLi-1 airway, Caco2, HT29 and T84 colorectal cells, gave similar results, with *E. coli* inducing IL-8 production. The viabilities of the NuLi-1 and Caco-2 cells were slightly reduced (8%) when exposed to the predators, while T84 viability remained steady. In no cases did the predatory bacteria induce actin rearrangement. These results clearly demonstrate the gentle natures of predatory bacteria and their impacts on human cells.

*Bdellovibrio*-and-like organisms (BALOs) are small Gram-negative δ-proteobacteria that are able to predate upon, enter and kill other Gram-negative bacteria^1^. They are ubiquitous in nature, ranging from terrestrial and aquatic environments^2^,^3^ to the gut of healthy human individuals^4^. Predatory bacteria employ multiple strategies to attack and/or kill their host bacteria^5^. Perhaps the best studied strategy involves them invading the periplasm of other Gram-negative bacteria, where they undergo a complex developmental cycle that terminates the prey cell viability and ultimately leads to the release of the predator’s progeny^6^. These organisms can predate upon a wide range of pathogenic bacterial strains in both planktonic^7^ and biofilm environments^8^,^9^,^10^,^11^,^12^. Moreover, the alleged dynamics of the predator-prey relationship that lead to a successful predation have recently been revealed^13^. Due to their unique bactericidal property, bacterial predators, including *Bdellovibrio bacteriovorus* and *Micavibrio aeruginosavorus*, are being considered as promising antibacterial agents, particularly against human pathogens^6^.

The number of infectious diseases caused by pathogenic bacteria that have become resistant to commonly administered antibiotics is a burning issue in medicine. As a result, increasingly more infections are becoming difficult to treat using traditional antimicrobial agents^14^,^15^,^16^,^17^. A case in point is the recent emergence of MCR-1 in China, which represents a breach in the effectiveness of the last group of antibiotics, polymyxins, by plasmid-mediated resistance^18^. Although currently confined to China, MCR-1 is likely to emulate and follow the spread of other global resistance mechanisms, such as carbapenem-resistant NDM-1 (New Delhi metallo-beta-lactamase-1) Gram-negative strains^19^. Another major difficulty in pathogen eradication is “biofilms”, a surface-adherent structure formed by various bacteria, including pathogens. Biofilms pose an additional hurdle as they can be up to thousand times more resistant to antimicrobial agents than their planktonic counterparts^20^,^21^. With all these concerns, it is highly demanding that some effective alternative strategies should be made available in order to fight multi-drug resistant (MDR) infections. One novel strategy is using specific bacteriophage as a biological therapeutic for controlling the infectingpathogen^22^,^23^.

Predatory bacteria, which have also been proposed as a potential agent to address multidrug resistant pathogens, present several advantages over the use of antimicrobial drugs^1^,^6^. A few recent studies assessed the susceptibility of human pathogens to predation by *Bdellovibrio* spp. and other predatory bacteria, showing that these predators successfully reduce pathogen numbers under laboratory conditions^7^,^24^. However, the safety and efficacy of these predatory bacteria, particularly in regards to their cytotoxicity or inflammatory response, have remained relatively unstudied until very recently, with only a couple of studies touching on these important issues^25^,^26^,^27^.

Moreover, although *in vitro* and *in vivo* tests have been performed with predatory bacteria in various mammals, such as mice, rabbits and guinea pigs^25^,^28^, even non-pathogenic Gram-negative bacteria can reportedly elicit an inflammatory response from cultured epithelial cells^29^,^30^,^31^. This response is thought to be a leading cause of inflammatory bowel diseases (IBD), including Crohn’s disease (CD) and ulcerative colitis (UC)^32^, within humans. Consequently, this study was undertaken to investigate the inflammatory and/or cytotoxic effect of predatory bacteria, which are both Gram-negative and non-pathogenic to humans^1^, with several different mammalian cell lines. Our study sheds light on interactions between predatory bacteria and human cells and provides novel insight into the potential use predatory bacteria as live antimicrobial agents.

## Results

### Effect of Predatory Bacteria on Murine Macrophage Raw 264.7 Cells

**Figure 1:**
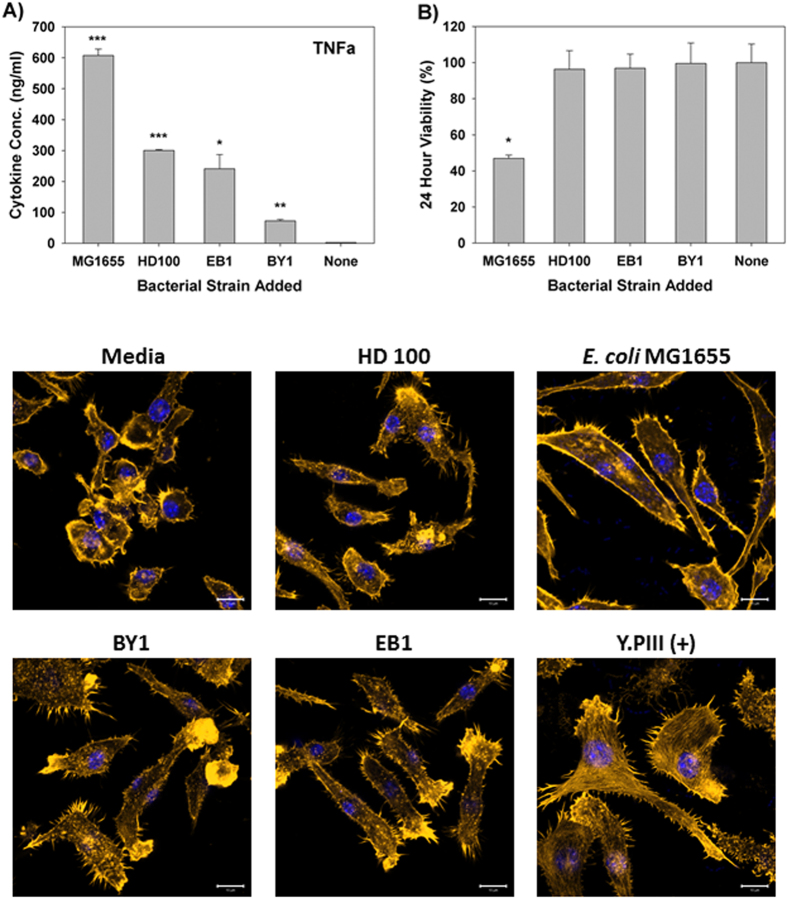
Response of RAW Macrophage 264.7 to predatory bacteria. (**A**) ELISA was performed for TNF-α within Raw 264.7 mouse monocyte-macrophage cell cultures during a direct exposure to the predatory bacteria. The MOI was 1230 predators per single mammalian cell. *E. coli* MG1655 (MOI = 111) was used as a representative Gram-negative strain. The concentration of the inflammatory protein was measured 6 hours post-inoculation of the bacteria (n = 3). (*p < 0.05; **p < 0.01; ***p < 0.001). (**B**) MTT assay showing Raw 264.7 cell viability with the indicated bacterial treatments after 24 hours. The results are presented relative to the media control (100% viable). The mean values from three independent experiments are shown with the error bars representing the standard deviation. (*p < 0.05). (**C**) Impact of a 4-hour pre-treatment of Raw 264.7 mouse macrophage cells with predatory bacteria (1230 predators per single mammalian cell) on actin filament rearrangement. Raw 264.7 cells were cultured on collagen-treated 8-well chambered coverslips and fixed after 4 hours. The nuclei and actin filaments were stained with DAPI (blue) and Rhodamine-Phalloidin (gold), respectively. Composite images show the nuclei (blue) and actin (gold, false color added for better resolution). The formation of prominent stress fibers throughout the cytoplasm was only seen when the Raw 264.7 cells were exposed to *Yersinia pseudotuberculosis* YPIII (MOI = 111). Images are representative of *n* = 3 independent experiments. Scale bar = 10 μm.

The criteria selected to evaluate the responses of the different mammalian cells to predatory bacteria in this study included the production of cytokines, their viability and any observable phenotypic changes. All exposures were performed with a bacteria-to-mammalian cell multiplicity of infection (MOI) of 111 for the non-predatory bacterial strains and 1230 for the predatory strains. This higher predator concentration was selected to demonstrate the safety of these microorganisms. As shown in Fig. 1A, treatment of the macrophage cells for six hours with the *Bdellovibrio bacteriovorus* strains, *i.e.*, *B. bacteriovorus* HD100 or BY1, induced significantly lower amounts of TNF-α (300 and 72 pg/ml, respectively) when compared to *E. coli* MG1655 (607 pg/ml). This *E. coli* strain was selected since it was the prey used for cultivating the predatory strains as well as a representative non-pathogenic Gram-negative γ-proteobacteria species. TNF-α induction with the third predatory strain, *Bacteriovorax stolpii* EB1, was likewise significantly lower (241 pg/ml) when compared to the *E. coli* strain. As noted above, the number of predatory bacteria per macrophage was approximately 10-fold higher than with *E. coli*. As such, the lower responses seen with the predators are all the more impressive given their significantly higher numbers present. ELISAs performed for IL-12 using the same samples found this pro-inflammatory cytokine was not induced by the predatory strains (Figure S1).

We next examined the 24 hour viability of the Raw 264.7 macrophage cells, as this may account for the reduced TNF-α or IL-12 production in response to the predatory bacteria. However, none of the predatory bacterial strains elicited a cytotoxic response, with macrophage viabilities greater than 96% when compared with the untreated media controls (Fig. 1B). In contrast, macrophage populations treated with *E. coli* MG1655 in parallel saw a 53% reduction in their viabilities (Fig. 1B), a result that is most likely due to overgrowth of this bacterial strain. Microscopic observation of the Raw 264.7 cells exposed to the predatory bacterial strains also revealed healthy macrophage populations in each case as no actin stress fiber formation was evident, a result that is in stark contrast to cells treated with *Y. pseudotuberculosis* YPIII strain (Fig. 1). These results suggest that predatory bacteria are only weakly immunogenic or active in inducing pro-inflammatory responses when exposed to immune cells like monocyte macrophages and that they are not cytotoxic.

### Effect of Predatory Bacteria on Lung Epithelial NuLi-1

**Figure 2:**
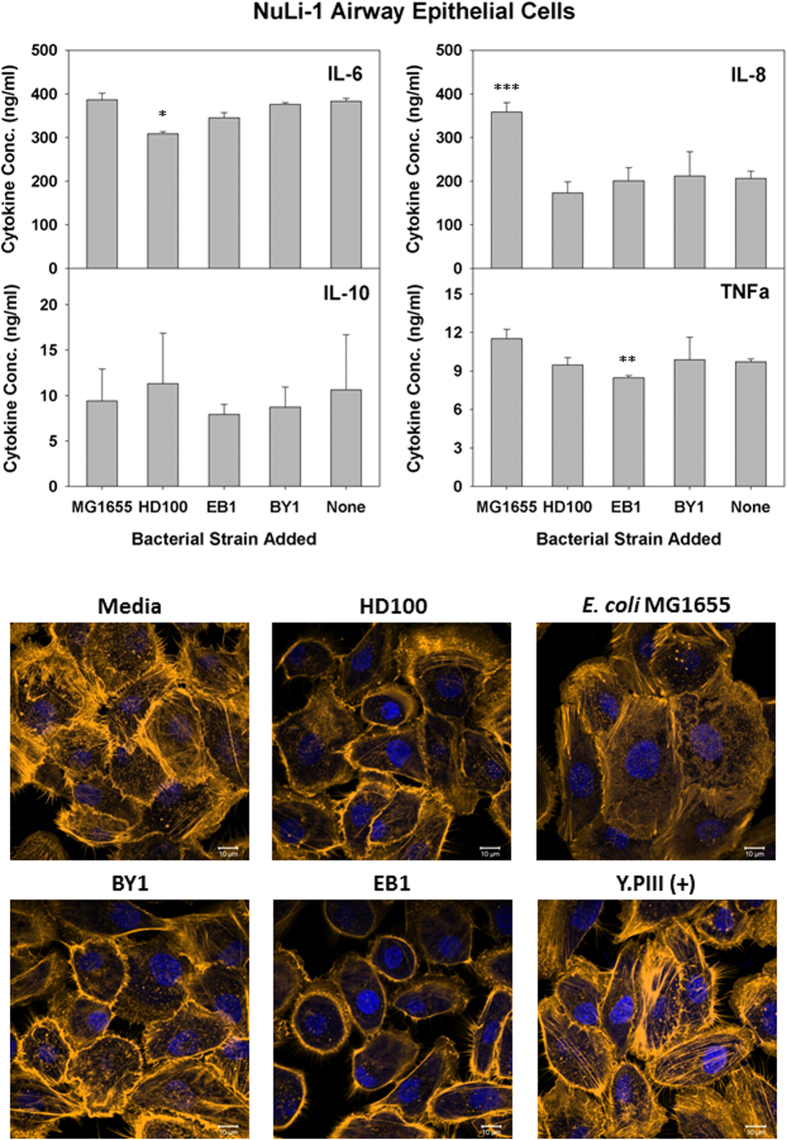
Induced inflammatory protein profile in response to predatory bacterial exposure to human alveolar epithelial NuLi-1. (Upper Panels) ELISA assays were performed for human pro-inflammatory cytokines IL-8 and TNF-α (left panels), the dual functioning IL-6 and the anti-inflammatory IL-10 (right panels) in response to predatory bacteria (1230 predators per single mammalian cell) in NuLi-1 human lung epithelial cell cultures relative to the untreated media controls. *E. coli* strain MG1655 (111 bacteria per single mammalian cell) was used as a representative Gram-negative strain. Inflammatory proteins were measured 6 hours post-inoculation of the bacteria (n = 3). The mean values from three independent experiments are shown with the error bars representing the standard deviation. (*p < 0.05; **p < 0.01; ***p < 0.001). (Lower Images) Predatory bacteria induced no early cytoskeletal changes in the alveolar epithelial NuLi-1 cells. Impact of a 4-hour pre-treatment of NuLi-1 cells with predatory bacteria (1230 predators per single mammalian cell) on actin filament rearrangement. NuLi-1 cells were cultured on collagen treated 8-well chambered coverslip and fixed after 4 hours. Nuclei were stained with DAPI (blue) and actin filaments with Rhodamine-Phalloidin (red), and fluorescent confocal microscopy was performed. The composite images show nuclei (blue) and actin (gold, false color added for better resolution). Images are representative of *n* = 3 independent experiments. Scale bar = 10 μm.

Given the promising results above, we next performed similar experiments with cells derived from different locations within the human body to determine if they interact differently with the predatory strains. Initially we chose to test NuLi-1 airway epithelial cells with all three predatory strains and *E. coli* MG1655. After treating the cells for 6 hours and collecting samples, ELISA tests were performed to measure several pro- and anti-inflammatory cytokines. As shown in Fig. 2, both IL-6 and IL-10 were not induced by the presence of the predatory bacterial strains. Production of two pro-inflammatory cytokines, IL-8 and TNF-α, was likewise unaffected by the predatory cells. For comparison, tests were also performed in parallel with *E. coli* MG1655, which elicited a strong IL-8 response from the NuLi-1 cells.

The gentle nature of the predatory strains towards human cells was further affirmed in the microscopic images of the exposed NuLi-1 cells in Fig. 2. These human cells appear unperturbed by the predatory strains, with no clear β-actin stress fiber formation or morphological changes obvious. Similar results were obtained when *E. coli* MG1655 was tested, although some of the cells were noticeably larger, suggesting that they may be experiencing a giant cell phenotype. In contrast, actin stress fiber formation was quite evident when *Y. pseudotuberculosis* YPIII was tested (Fig. 2).

### Effects of Predatory Bacteria on Intestinal Epithelial Cells

**Figure 3:**
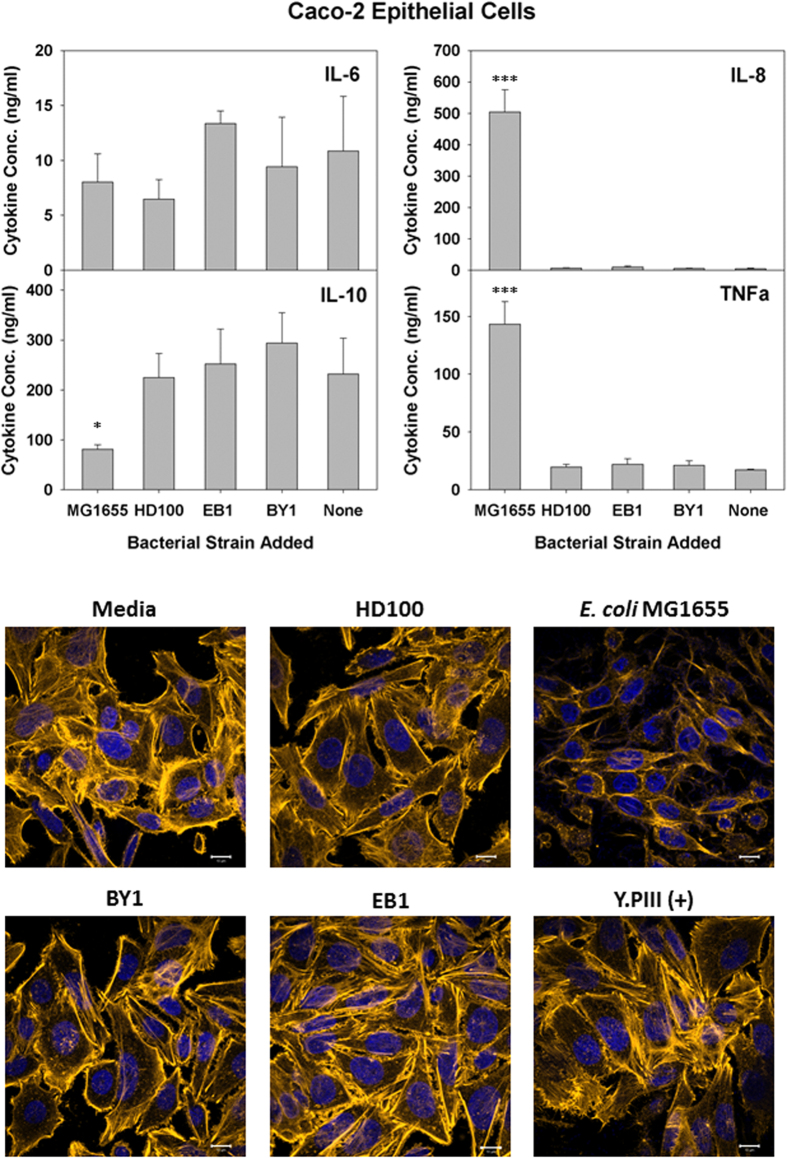
Response of Caco-2 cells to predatory bacteria. (Upper Panels) ELISA assays were performed for human pro-inflammatory cytokines IL-8 and TNF-α (left panels) the dual functioning IL-6 and the anti-inflammatory IL-10 (right panels) in response to predatory bacteria (MOI = 1230) in Caco-2 cultures relative to the untreated media controls. *E. coli* strain MG1655 (MOI = 111) was used as a representative Gram-negative strain. Inflammatory proteins were measured 6 hours post-inoculation of the bacteria (n = 3). The mean values from three independent experiments are shown with the error bars representing the standard deviation. (*p < 0.05; ***p < 0.001). (Lower Images) Predatory bacteria induced no early cytoskeletal changes in Caco-2 cells. Impact of a 4-hour pre-treatment of Caco-2 cells with predatory bacteria (MOI = 1230) on actin filament rearrangement. Caco-2 cells were cultured on collagen-treated 8-well chambered coverslips and fixed after 4 hours. Nuclei were stained with DAPI (blue) and actin filaments with Rhodamine-Phalloidin (red), and fluorescent confocal microscopy was performed. The composite images show nuclei (blue) and actin (gold, false color added for better resolution). Images are representative of *n* = 3 independent experiments. Scale bar = 10 μm.

As the results above show all three predatory bacterial strains caused no obvious harm to NuLi-1 airway epithelial cells, we next analyzed their impacts on various intestinal epithelial cells (IECs). As with the NuLi-1 cells, ELISA’s were performed once more to determine the concentrations of the four pro- and anti-inflammatory cytokines produced in polarized T84 cell cultures (Figure S2) after a 6 hour exposure (Fig. 3). The cytokine responses from the T84 colorectal cells were basically identical to those from NuLi-1 cell cultures, namely that only IL-8 was induced and this from an exposure to *E. coli* MG1655. None of the predatory bacterial strains tested significantly induced the production of the cytokines tested with this cell line (Fig. 3). Images of the T84 cells taken after a 4-hour exposure likewise found no clear morphological changes resulting from the predatory bacteria.

**Figure 4:**
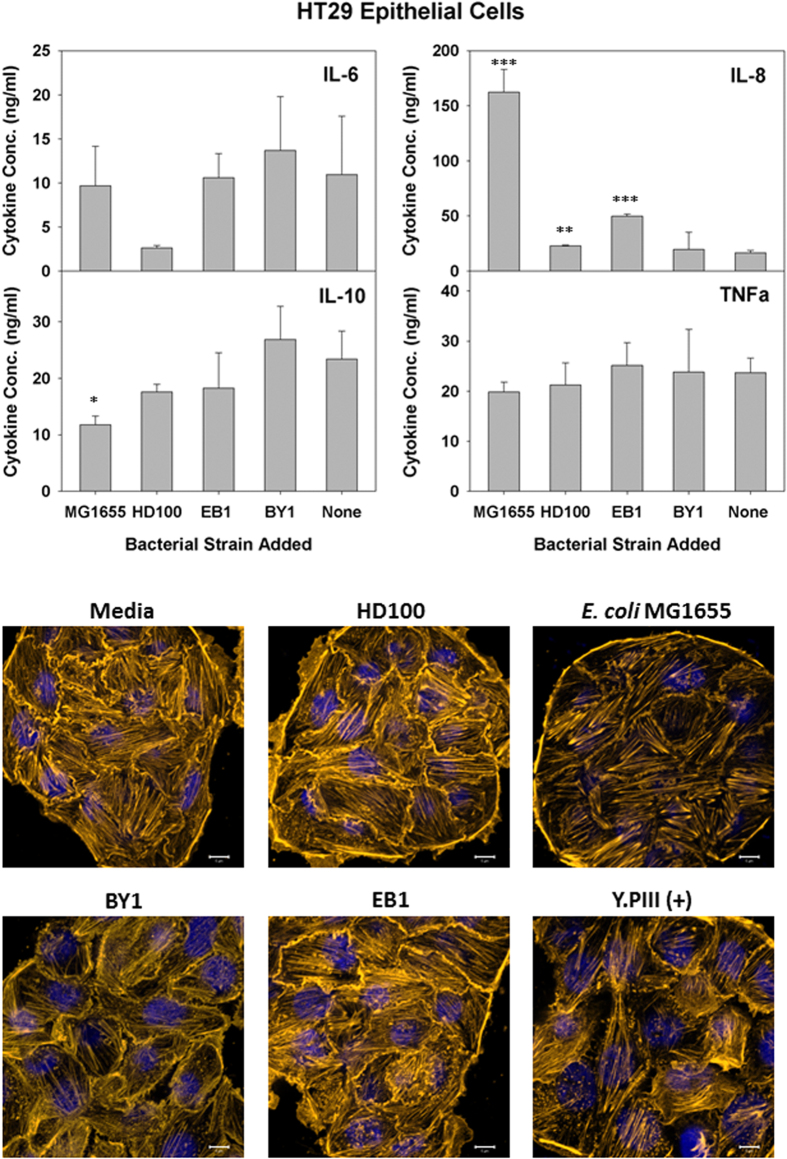
Response of HT29 cells to predatory bacteria. (A) ELISA assays were performed for human pro-inflammatory cytokines IL-8 and TNF-α (left panels) the dual functioning IL-6 and the anti-inflammatory IL-10 (right panels) in response to predatory bacteria (MOI = 1230) in HT29 cultures relative to the untreated media controls. *E. coli* strain MG1655 (MOI = 111) was used as a representative Gram-negative strain. Inflammatory proteins were measured 6 hours post-inoculation of the bacteria (n = 3). The mean values from three independent experiments are shown with the error bars representing the standard deviation. (*p < 0.05; **p < 0.01; ***p < 0.001). (Lower Images) Predatory bacteria induced no early cytoskeletal changes in HT29 cells. Impact of a 4-hour pre-treatment of HT29 cells with predatory bacteria (MOI = 1230) on actin filament rearrangement. HT29 cells were cultured on collagen-treated 8-well chambered coverslips and fixed after 4 hours. Nuclei were stained with DAPI (blue) and actin filaments with Rhodamine-Phalloidin (red), and fluorescent confocal microscopy was performed. The composite images show nuclei (blue) and actin (gold, false color added for better resolution). Images are representative of *n* = 3 independent experiments. Scale bar = 10 μm.

**Figure 5:**
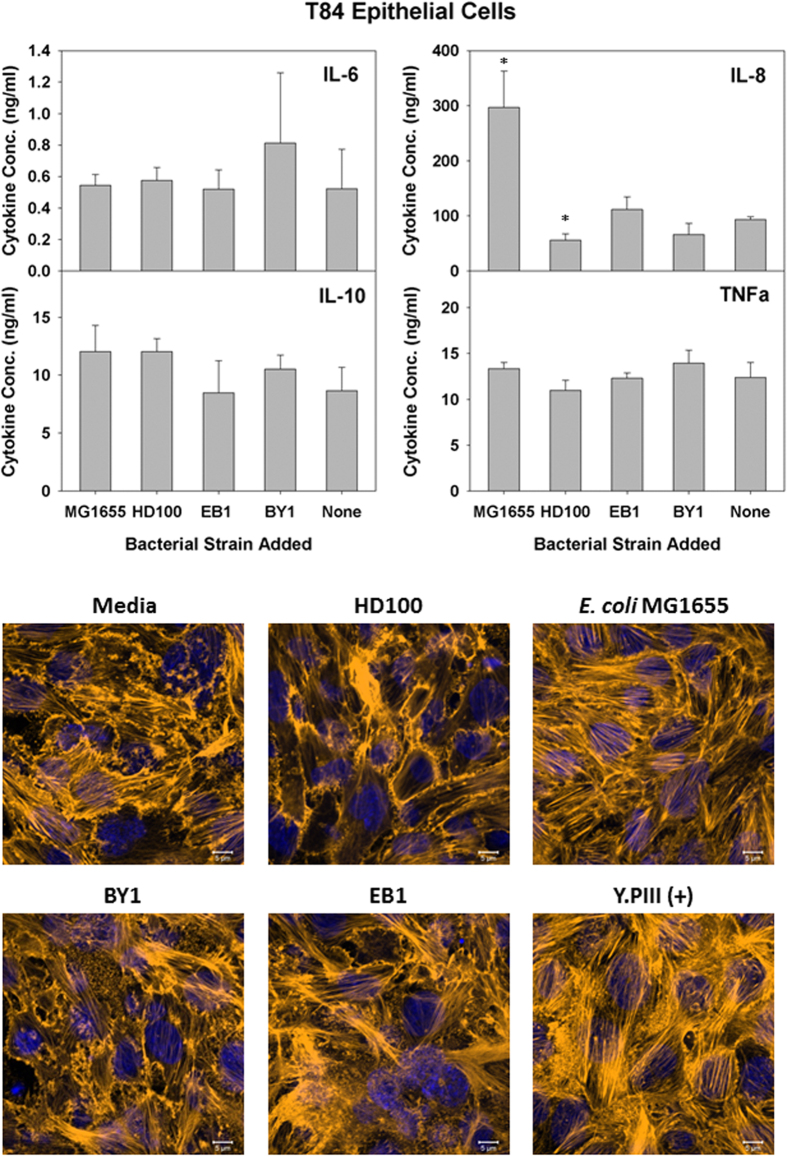
Response of T84 cells to predatory bacteria. (Upper Panels) ELISA assays were performed for human pro-inflammatory cytokines IL-8 and TNF-α (left panels) the dual functioning IL-6 and the anti-inflammatory IL-10 (right panels) in response to predatory bacteria (MOI = 1230) in T84 cultures relative to the untreated media controls. *E. coli* strain MG1655 (MOI = 111) was used as a representative Gram-negative strain. Inflammatory proteins were measured 6 hours post-inoculation of the bacteria (n = 3). The mean values from three independent experiments are shown with the error bars representing the standard deviation. (*p < 0.05). (Lower Images) Predatory bacteria induced no early cytoskeletal changes in T84 cells. Impact of a 4-hour pre-treatment of T84 cells with predatory bacteria (MOI = 1230) on actin filament rearrangement. T84 cells were cultured on collagen-treated 8-well chambered coverslips and fixed after 4 hours. Nuclei were stained with DAPI (blue) and actin filaments with Rhodamine-Phalloidin (red), and fluorescent confocal microscopy was performed. The composite images show nuclei (blue) and actin (gold, false color added for better resolution). Images are representative of *n* = 3 independent experiments. Scale bar = 10 μm.

Similar results were seen with two additional IECs, *i.e.*, HT29 and Caco-2 cells (Figs 4 and 5, respectively). The cytokine production profile for HT29 cells was nearly identical as that for T84, except that *B. bacteriovorus* HD100 and *B. stolpii* EB1 both led to slight, yet significant, inductions of IL-8 (Fig. 4). Given that the number of predatory bacteria added was ten-fold higher than that for *E. coli* MG1655, these responses are mild by comparison. The cytokine results for Caco-2 were also similar with T84 except that TNF-α production was also strongly induced in the former cell line by *E. coli* MG1655. For all three IECs tested, the IL-10 levels remained constant or were reduced during exposure to the various bacterial strains. Once more, the predatory microbes had no obvious impact on the HT29 and Caco-2 IEC morphologies (Figs 4 and 5, respectively).

### Human Epithelial Cell Viabilities

**Figure 6:**
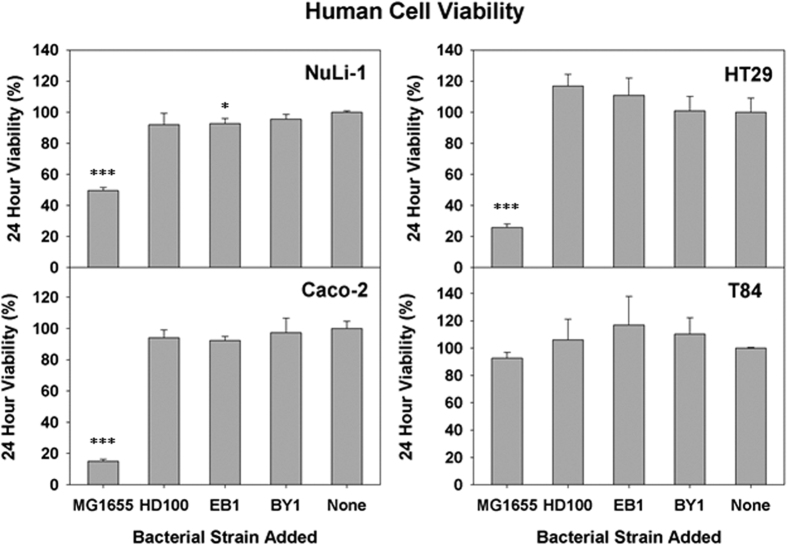
Viability of NuLi-1, Caco-2, HT29, and T84 cells in response to the indicated bacterial treatments. MTT assays were performed to measure the viability of the four human cell lines 24 hours after an exposure to the predatory bacterial strains (MOI = 1230). *E. coli* strain MG1655 (MOI - 111) was used as a representative Gram-negative strain. The results are shown relative to the media alone samples. The mean values from three independent experiments are shown with the error bars representing the standard deviation. (*p < 0.05; ***p < 0.001).

Using the same bacteria-to-human cell MOIs, *i.e.*, 1230:1 and 111:1 for the predatory strains and *E. coli* MG1655, respectively, we next determined the impact each strain had on the 24 hour viability of the NuLi-1 cell cultures and IECs. For NuLi-1 cultures, the viability in the presence of the predatory strains was ≥90%, but dropped by 50% when exposed to *E. coli* MG1655 (Fig. 6). Of the IECs, the T84 cell cultures were the most robust epithelial cell tested since *E. coli* MG1655 reduced the viability by only 7% (Fig. 6). By comparison, HT29 and Caco-2 were much more susceptible to *E. coli* MG1655, with viability losses of 74% and 85%, respectively.

**Figure 7:**
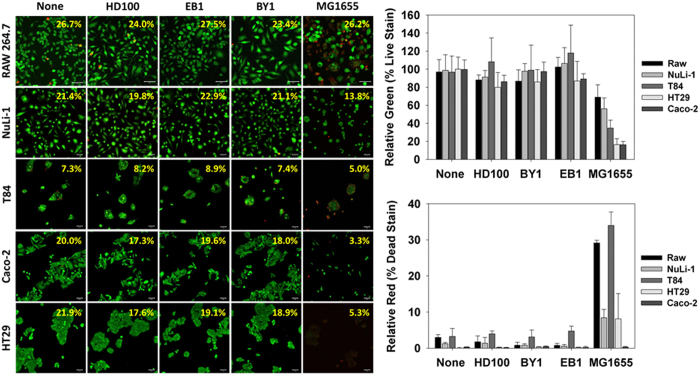
Live/dead analysis of RAW 264.7, NuLi-1, Caco-2, HT29, and T84 cells in response to the indicated bacterial treatments. (Left Images) Live and dead cells were quantified by using two dyes, calcein AM and ethidium homodimer-1 (EthD-1), respectively. Confocal images of each cell line grown in monolayers 24 hours after exposure to the predatory bacterial strains (MOI = 1230) or *E. coli* strain MG1655 (MOI = 111) (Scale Bar = 50 μm). The numbers given in the upper right-hand corner of each image is the percent surface coverage for the cells obtained from the ImageJ analyses. (Right Panels) Calculated percent of live (green) and dead (red) cells in each sample based upon the ImageJ analyses. The results are the mean values from five independent samples with the error bars representing the standard deviation.

These results are in agreement with the live/dead images obtained for each cell type, including the Raw 246.7 cells (Fig. 7). For each cell type, the live cells fluoresce green while those that are dead or dying are red or yellow in color. It is clear from the images that *E. coli* MG1655 significantly impacted all of the cells tested in this study, leading to either a substantial loss in viability (Raw 246.7) or a reduced surface coverage (epithelial cells). For each of the predators, however, the results were basically identical with those in the media control. This is further confirmed in the ImageJ analyses where the relative number of green pixels, which correspond to the live cells, decreased when the cells were exposed to *E. coli* MG1655 but not the predatory cells.

## Discussion

With the current state of emerging multidrug-resistant pathogens, research into areas of microbiology and infectious disease that show promise towards the development of antimicrobial agents or strategies is rapidly increasing in significance. One novel approach that might hold the potential to treat antibiotic resistant infections is predatory bacteria^6^. *Bdellovibrio* strains, and other predatory bacteria, have long been proposed as a future alternative for antimicrobial therapy, particularly for external use such as in infected skin wounds^1^. The gentle nature of predatory bacteria towards human cells was hinted at in a previous study where epithelial cells exposed to a human pathogen were protected by the activity of *B. bacteriovorus* HD100^33^. Furthermore, their safety in mammalian systems has recently been addressed in an *in vivo* mouse model^25^. However, their effect on humans is an area of research that has not been thoroughly examined, either *in vitro* or *in vivo*.

In this study, we corroborated the effect of predatory bacteria on both cultured murine macrophage and human epithelial cell lines. Initially, mouse Raw 264.7 monocyte-macrophage cells were used as a representative immune cell to determine how they respond to three different predatory bacterial isolates, two *Bdellovibrio* spp. (HD100 and BY1) and one *Bacteriovorax* spp. (EB1). Amongst these three bacterial strains, BY1 and EB1 were both isolated from aquatic environments and identified through 16 s genotyping and phylogenetic analyses (Figure S3). The ability of each of these strains to elicit inflammatory responses from the macrophage cultures was determined alongside *E. coli* MG1655, a common lab strain that was selected in this study as a representative Gram-negative enterobacterium. Our results found lower TNF-α and IL-12 cytokine induction levels with the predatory bacteria, which suggest that they likely have a lower endo-toxic activity associated with their cell membrane components^34^,^35^. This is quite in line with a study performed with human mononuclear cells (hMNC) where the authors showed that purified lipid A and LPS from *B. bacteriovorus* HD100 was less immunogenic than those from an *E. coli* K-12 strain^36^. Their study identified some basic differences in the cell wall components between host dependent predatory bacteria and their natural prey *E. coli* strain. Thus, it appears that the membrane components of both *Bdellovibrio* and *Bacteriovorax* spp. are inert, or at least less active in inducing pro-inflammatory innate immune responses from cultured Raw 264.7 murine macrophage cells.

Another recent study, performed by Shatzkes, K. *et al*.^25^, reported modest cytokine induction levels shortly after an intravenous injection of predatory bacteria into live mice^25^. These induced levels dropped to baseline readings within 18 hours. One significant difference between their study and ours is the level of TNF-α detected, which was higher here. This discrepancy, however, can be explained by the direct exposure of the predatory bacteria to these immune cells in this study rather than their dissemination throughout the circulatory system of a live mouse, which would disperse them throughout various anatomical locations. The lack of any cytotoxic nature against Raw 264.7 monocyte-macrophage cells suggests that the predatory strains lack of any secretory or membrane bound cytolytic proteins. Microscopic data also found no obvious physical or cytoskeletal alteration of the Raw 264.7 monocyte-macrophage cells upon exposure (Figs 1 and 7).

Our initial observation that predatory bacteria are less immunogenic and less cytotoxic to a professional immune cell further supports the idea that these bacteria are not harmful to eukaryotic cells. However, as this finding may not correlate with the responses from human cells, several cell lines from different tissues were selected for evaluation. As one of the earliest cytokine to be secreted by epithelial cells, IL-8 is typically induced in response to bacterial entry^37^ or foreign antigens like flagellin^38^ or invasion^39^ proteins through toll-like receptor-mediated NF-κB and the mitogen activated protein kinase (MAPK) pathway^40^. This was the case for *E. coli* MG1655, where an exposure to this bacterium led to an increased IL-8 production from NuLi-1 cells. As the pro-inflammatory cytokines IL-8 and TNF-α are a series of immunoregulatory molecules that control the pro-inflammatory cytokine response^41^,^42^, we next analyzed the levels of two additional cytokines, *i.e.*, IL-6 and IL-10. Whereas IL-10 is classified as anti-inflammatory cytokine, IL-6 is cell-type dependent and can function either anti-inflammatory or pro-inflammatory^43^. Interestingly, the basal level expression of IL-6 in NuLi-1 culture was much higher than the other cell lines tested, even for the non-treated medium control. The concentration of this cytokine remained high in all replications of this experiment, and, as such, appears to be a natural phenomenon of these alveolar epithelial cells. In support of this, it was shown previously that NuLi-1 cells are good producers of IL-6, which in turn activates alveolar macrophages to fight infection^44^. Although this higher expression may act to mask the response from these cells, IL-6 production levels were not induced by either *E. coli* MG1655 or the predatory strains. Similar results were seen with both IL-10 and TNF-α.

Another group reportedly found predatory bacteria within the human gut^4^ and, thus, we also tested the impact of an exposure with several IECs, including T84 cells. As reported previously, a basolateral adhesion of the bacteria with differentiating T84 cells is required to induce pro-inflammatory IL-8^45^. Consequently, polarized T84 cells were generated in trans-well inserts to permit an exposure at the basolateral surface. With T84 cells, no IL-8 induction was seen with the predatory strains while the addition of *E. coli* elicited a substantial production of this cytokine. Similar results were also seen in another study where a human corneal-limbal epithelial cell line responded similarly when exposed to either *Bdellovibrio* spp. or *Pseudomonas aeruginosa* PAO1^46^. Furthermore, tests with two additional IECs, *i.e.*, HT29 and Caco-2 cells, gave basically identical results. Only with HT29 cells did the predatory strains *B. bacteriovorus* HD100 and *B. stolpii* EB1 elicit an IL-8 response. However, although the impact was significant, it was still very mild when compared with the response resulting from an exposure to *E. coli* MG1655. Given the much higher addition of predatory cells (11.1-fold higher) than *E. coli*, this weaker response further affirms the results of Schwudke *et al*.^36^ that predatory bacteria are not strongly immunogenic.

None of the IECs showed increasedIL-6, IL-10 or TNF-α production levels when exposed to the predatory bacterial strains, further affirming that these strains elicit only a minimal inflammatory response from these human cells. In contrast, *E. coli* MG1655 led to a strong TNF-α production from Caco-2 cells. Exposure to this bacterium also led to a significant reduction in the IL-10 cytokine levels within Caco-2 and HT29 cultures. This loss, however, may be attributed in part to the impact *E. coli* MG1655 had on the viabilities of both human cell lines as both were very susceptible to this strain (Fig. 6). Their sensitivity was further verified in the live/dead experiments (Fig. 7), where the populations of both Caco-2 and HT29 were nearly completely eradicated by *E. coli* MG1655. In contrast, the live/dead results given in Fig. 7 support the MTT viability results (Fig. 6) as both show all three predatory strains are not harmful to the human cells tested.

Although the results affirm that predatory bacteria do not negatively impact the health of the mammalian cells tested here, it is clear that the responses varied somewhat between the cell lines. LPS is recognized by the immune system as a marker for the detection of bacterial and pathogen invasion, a response that is responsible for the development of inflammatory system^47^. Due the nature of the LPS-host defense interactions, dissimilarities in the inflammatory and immunological responses from the different human cell lines tested are to be expected. For example, the biophysical properties of LPS-binding proteins, like the MD-2 and Toll-like receptor 4 complex (MD-2–TLR4), and their mode of interaction with LPS determine the ability of LPS to activate the immune response^48^. Furthermore, not all cell lines produce every cytokine in response to bacterial invasion or their secretory proteins. With this in mind, we selected mammalian tissue culture models, like macrophages and IEC’s, which are known to secrete most, if not all, of the cytokines assayed for in this study. That being said, no single cell line can be classified as an absolutely perfect system for evaluating predatory bacteria and, as such, to minimize this natural limitation, an array of cells was assayed in this study.

Although the predatory bacteria did not induce cytokine production from or reduce the viabilities of the mammalian cells tested, they may still be detrimental. To delve deeper into some potential responses that may result, we next looked for the formation of any stress fibers during an exposure. The driving force of cellular motility, cytokinesis and vesicular trafficking processes depends on the dynamic remodeling of the actin cytoskeleton through assembly, disassembly and organization of actin filaments into functional networks. It is known that Rho family small GTPases regulate cytoskeletal dynamics and that Rho activity is required for the formation of actin stress fibers and focal contacts, which are known to be formed when bacteria invade human cells. Thus, actin rearrangement is an important physiological process that can be used to evaluate a eukaryotic cells response during the course of bacterial exposure or internalization. When the human cells were exposed to the invasive pathogen *Yersinia pseudotuberculosis* YPIII exhibited extensive actin stress fiber formation. This was expected as this *Y. pseudotuberculosis* YPIII is known to elicit this response^49^ and for this reason was used as a positive control. In stark contrast, cells exposed to the predatory bacteria displayed no apparent increase in actin stress fiber formation. This was true for each of the predatory strains, as well as *E. coli* MG1655. The lack of any response implies that predatory bacterial strains are non-invasive with respect to human cells.

In conclusion, this study sheds light on and partially alleviates a major concern related with predatory bacterial research, namely the safety issue connected with their potential use in humans. Our findings demonstrate that a high-level exposure to predatory bacteria is not harmful to human cells *in vitro*. This is particularly supported by the absence of any strong phenotype when the three human cells were exposed to the predatory strains, either in terms of their cytokine production levels, losses in viability or the formation of actin stress fibers. However, further studies are needed to fully establish the gentle nature of predatory bacteria towards eukaryotic hosts as all the tests here were performed *in vitro* using cell lines and cultures and may not reliably replicate the responses that would be seen within, say, a human host. The results presented here, though, help pave the way for future studies focusing on predatory bacteria and their *in vivo* efficacy against bacterial pathogens.

## Methods

### Bacterial Strain Isolation, Identification and Growth

Wild-type host dependent predatory *Bdellovibrio bacteriovorus* HD100 was purchased from the German Collection of Microorganisms and Cell Cultures (DSMZ). *E. coli* MG1655 was used as both the prey for cultivation of the predatory strains as well as a standard Gram-negative non-pathogenic bacterial strain for these experiments. *Yersinia pseudotuberculosis* strain YPIII (p-) was obtained from BEI Resources (Manassa, VA, USA).

The other predatory bacteria, *B. bacteriovorus* BY1 and *Bacteriovorax stolpii* EB1 were isolated from environmental samples following methods described elsewhere^50^ with slight modifications. Briefly, 10 grams of underwater sediment was collected from the Taehwa River near Ulsan, Republic of Korea and mixed with 20 ml of HEPES buffer containing Mg^2+^ and Ca^2+^ with swirling onto a dancing benchtop rotator (Twister, Vison Scientific, Korea) for two hours. The mixture was then centrifuged at 2900 × g for 5 minutes using a benchtop centrifuge machine (Eppendorf 5430R, Germany). The supernatant was collected, filtered through a 0.45 micron filter (Millex-Millipore Ltd, USA) and 5 ml was mixed with 1 ml of washed *E. coli* MG1655 in the same HEPES buffer (OD = 2). The predator-prey mixture was then incubated in a 30 °C shaking incubator for 2 hours at 200 rpm. Afterwards, 2 ml were mixed with 10 ml of DNB top agar supplemented with the salts and poured onto a bottom agar plate as described previously. Plates were incubated at 30 °C until clear plaques could be visualized. These plaques were then collected and sub-cultured with freshly prepared prey (*E. coli*).

The newly isolated predators were then identified via 16 s rDNA sequencing with the universal primers 27F/1492R primer set^51^ followed by phylogenetic analysis with MEGAversion 6 software^52^. Predatory and prey bacteria were cultured and maintained in DNB (dilute nutrient broth 1/10 dilution of NB containing both 2 mM CaCl2 and 3 mM MgCl2) and LB (10 g tryptone, 5 g NaCl, and 5 g yeast extract per liter) medium, respectively, according to the protocol describedpreviously^53^.

### Cell Lines, Cultures and Construction of Monolayers

Murine monocyte macrophage Raw 264.7 cells, human colon carcinoma T84 cells and Caco2 were cultured in T75 flasks in Dulbecco’s Modified Eagles Medium (MEM, 1:1 with sodium bicarbonate (Life Technologies, USA)). DMEM with sodium bicarbonate was used for growth of HT29. Each media was substituted with L-glutamine, 10% heat inactivated fetal bovine serum and 100 μg/ml Normocin (Invivogen, USA). Human primary alveolar epithelial NuLi-1 cells were cultured similarly in Bronchial Epithelial Growth Medium (BEGM) supplemented with 100 μg/ml Normocin (Invivogen, USA) without FBS. Each cell line was cultured under 5% CO_2_ in a water-jacketed CO_2_ incubator until they were confluent. For the ELISA tests, approximately 4 × 10^4^ cells were seeded in a well of regular 12 well tissue culture plates (SPL Life Sciences, South Korea). T84 intestinal epithelial cells were seeded onto 12 mm Costar^®^ polystyrene (Corning Inc. USA) trans-well plates (0.3 μm pore size, 0.33 cm^2^ insert diameter, 10^6^ cells/cm^2^ and allowed to grow for two weeks to obtain polarized epithelial cells. The trans-well inserts were equilibrated in culture medium for at least 2 hours before seeding. The media in each plate was changed as needed.

### Infection Protocols

The bacterial suspensions used to treat the various mammalian cells were prepared from freshly grown bacterial culture as described elsewhere^45^. For the predatory bacterial strains, 24 hour cultures grown on *E. coli* MG1655 were used. These cultures were first filtered through 0.45 μm syringe filter (Millex-Millipore Ltd, USA) to remove residual prey and the filtrates were concentrated by spinning them down at 10,600 × *g* for 5 minutes, washed four times with sterile HEPES (pH 7.4) and finally suspended in the appropriate medium at a concentration of 2.5 ± 0.74 × 10^7^ cells/ml. The media in the wells with the human cells was then replaced with 1 ml of this media containing the predatory strains.

For the *E. coli* strain, an overnight culture of *E. coli* MG1655 was diluted 100-fold in fresh LB and grown for 3 hours at 37 °C at 200 RPM, after which the cells were collected by centrifugation at 10,600 × *g* for 5 minutes and washed twice in HEPES. After determining the OD, appropriate dilutions of the bacteria in the appropriate media were prepared before initiating the exposure. The average predatory bacterium-to-human cell ratio used in the exposures was 1230 ± 371:1 while the *E. coli*-to-human cell ratio was approximately 111 ± 17:1. The actual number of bacteria administered was determined by serially diluting and plating to obtain the PFU/CFU numbers at 30 °C.

For the Raw 264.7, Caco2, HT29 and NuLi-1 cells, the cells were allowed to form monolayers for 24 hours in regular 12 well plates. Afterwards the medium was removed, the cells were washed with DPBS and then they were equilibrated with serum- and antibiotic-free medium for at least 2 hours before addition of the bacteria. Likewise, the polarized T84 monolayers grown on Costar^®^ polystyrene trans-well plates were washed two times with DPBS and incubated in medium containing heat-inactivated fetal bovine serum without antibiotics for 1 to 2 h. After equilibration, the bacteria were added to the lower chamber to allow basolateral contact. After six hours of exposure, the cell culture supernatant samples (apical in case of trans-well plates) were collected and centrifuged at 3,900 × *g* for 20 min to pellet any residual bacteria and cells before cytokine measurement.

### Cytokine Assays

After collection of the cell culture suspensions, the samples were stored at −20 °C until needed. The cytokine levels in each of samples were measured using enzyme-linked immunosorbent assay (ELISA). Mouse TNF-α and IL-12 ELISA’s were performed with the samples collected from Raw 264.7 macrophage cultures. In a similar manner, the human pro-inflammatory IL-8 and TNF-α, the dual functioning IL-6 and the anti-inflammatory IL-10 ELISAs were performed using kits from R&D systems Inc. (USA) according to the manufacturer’s protocol. The values are expressed as pg/ml.

### Cytotoxicity Assays

The impact of the bacteria on the 24 hour viability of the cultured eukaryotic cells was also determined with the MTT assay. After the 24 hour exposure, approximately 5 μg/ml of 3-(4,5-dimethyl-2-thiazolyl)-2,5-diphenyl-2H-tetrazoliumbromide (MTT reagent, Life Technologies, USA) was added to each well and the plates were incubated at 37 °C for 2–4 hours in the dark. Subsequently, the media was thoroughly removed, about 400 μl of DMSO was added to each well and the plate was incubated with shaking (150 rpm) at room temperature for 15 minutes to allow the color to develop. The OD was measured at 540 nm and used as a proxy for the viability of the cultured mammalian cells.

### Immunostaining and Confocal Microscopy

To inspect the cells for any morphological changes induced by exposure to the bacteria, confluent monolayers were prepared on Labtek™ II chambered cover glass (Nunc, Germany). Before treatment, *i.e.*, addition of the bacteria, the mammalian cells were serum starved for at least 20 hours. Cells were then treated with the predatory bacteria (MOI 1230:1) and *E. coli* and *Yersinia pseudotuberculosis* strain YPIII (MOI 111:1) for 3 h. Subsequently, the mammalian cells were washed with DPBS and fixed with 3.7% paraformaldehyde (in PBS) for 20 min at room temperature. After fixing, the cells were washed with PBS and permeabilized with 0.1% Triton X-100 (Sigma Aldrich, USA) in PBS for 5 min. The actin cytoskeleton was then stained with a phalloidin-rhodamine solution (0.5 μg/ml in DPBS; Invitrogen, USA) for 30 min at room temperature. After washing the cells with DPBS, the cellular DNA was counter-stained with DAPI (1 μg/ml in PBS)(Life Technologies, USA) for 5 min at room temperature. These cells were then visualized using a laser confocal microscope LSM 700^®^ and the captured images processed using Zen software (Carl-Zeiss, Germany).

### Live/Dead Imaging and quantification

Live, dead and total cells were quantified by using two dyes, calcein AM and ethidium homodimer-1 (EthD-1), to stain the cells. The cells were prepared by seeding 2 × 10^4^ cells/well in 12 well plates. After 24 hours cells were treated with either media alone, a predatory strain or *E. coli* MG1655 for 24 hours as mentioned above. The cells were then washed gently with 1X Dulbecco’s phosphate-buffered saline (DPBS) at room temperature.

To perform the assay, 400 μL mixture of the Live/Dead Assay reagents (containing approximately 1 μM calcein AM and 2 μM EthD-1 (Life Technologies, USA)) were added to each well. The cells were incubated at room temperature for 10 minutes and subsequently observed under a confocal microscope (LSM 700; Carl Zeiss, Germany). For each condition, five independent fluorescent images were taken and analyzed using ImageJ software (http://rsb.info.nih.gov/ij/). The surface coverage and the relative green and red portions for each cell line and treatment were determined using the ImageJ data.

### Statistical Analysis

The data is expressed as the mean with the standard deviations (SDs). An unpaired Student’s t test was used to analyze the data. *P*-values of <0.05 were considered statistically significant. All the experiments were repeated at least three times for error analysis.

## Additional Information

**How to cite this article**: Monnappa, A. K. *et al*. Investigating the Responses of Human Epithelial Cells to Predatory Bacteria. *Sci. Rep.* **6**, 33485; doi: 10.1038/srep33485 (2016).

## Supplementary Material

Supplementary Information
