# Supplementary material for: Investigating the Responses of Human Epithelial Cells to Predatory Bacteria
Source: Sci Rep. 2016 Sep 15;6:33485. doi: 10.1038/srep33485 (PMC5024164; doi:10.1038/srep33485)
Supplement: Supplementary Information [file srep33485-s1.pdf]

Supplemental Information for

Investigating the Inflammatory and Phenotypic Responses of Multiple Cultured Human Epithelial Cells to Predatory Bacteria

Wasimul Bari#, Ajay K. Monnappa#, Seong Yeol Choi and Robert J. Mitchell [*]

School of Life Sciences, Department of Biological Sciences, Ulsan National Institute of Science and Technology, 689-798

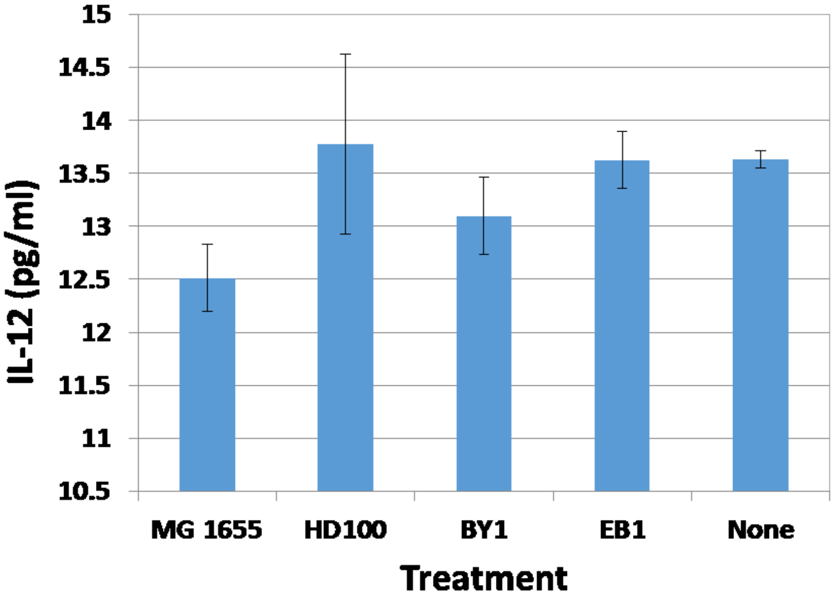

Supplementary Figure 1. Pro-inflammatory cytokine IL-12 induction in response to predatory bacterial exposure to mouse macrophage Raw 264.7 cells. ELISA was performed for mouse IL-12 in response to direct exposure of predatory bacteria (1000 predators per single mammalian cell) *B. bacteriovorus strain* HD100, BY1 or *Bacteriovorax stolpii* strain EB1 in Raw 264.7 cultured mouse monocyte-macrophage cell line relative to untreated media control (DMEM:F12). *E.coli* strain MG1655 (100 bacteria per single mammalian cell) was used as a representative gram negative strain. Inflammatory protein was measured at 6 hours post-inoculation of bacteria (n=3). Data shown from three independent experiments. Each bar represent mean ± standard deviation.

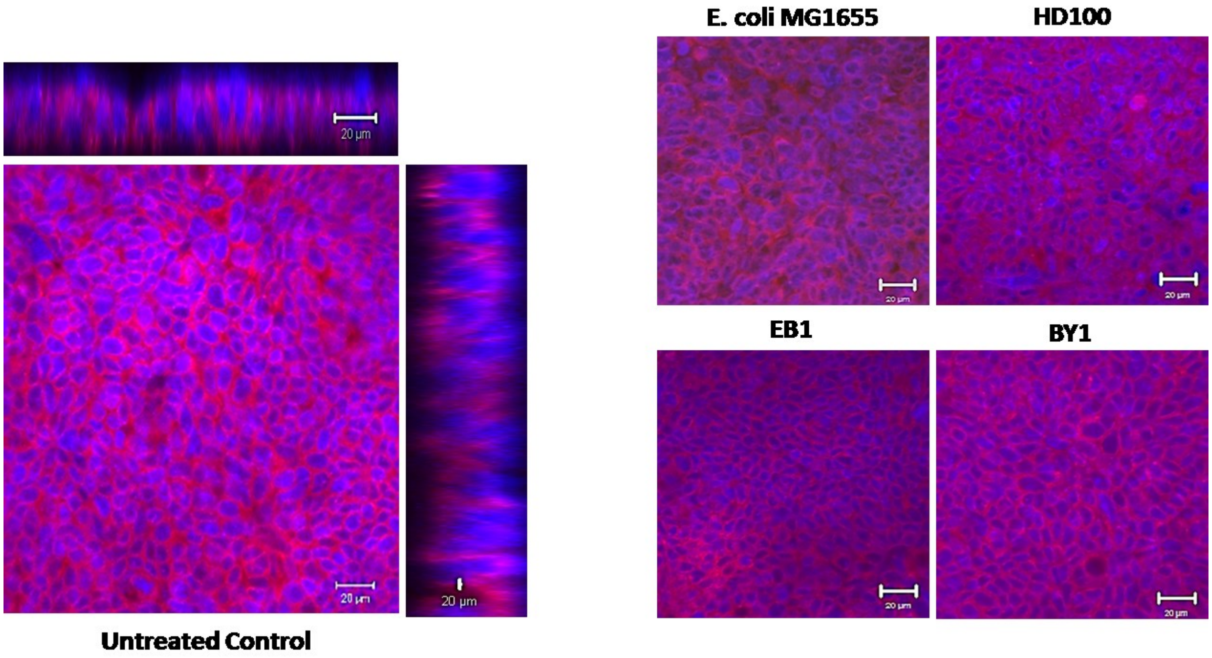

Supplementary Figure 2. Confocal immunofluorescent microscopic image of polarized T84cells. (A) Human intestinal epithelial T84 cells were cultured on trans-well filters (12mm, 3µm pore size) for 2 weeks in order to obtain polarized cells. Cells were subjected to basolateral infection by predatory bacteria (1000 predators per single cell) *B. bacteriovorus strain* HD100, BY1 or *Bacteriovorax stolpii* strain EB1 and *E.coli* strain MG1655 (100 bacteria per single mammalian cell) for 6 hours. **(B)** Microscopic image showing the polarity of T84 cells in multi-plane z-stack. For imaging cell were stained with CellTracker Red CMTPX (Red) and nucleus were counterstained with DAPI (Blue). Scale bar=20µm

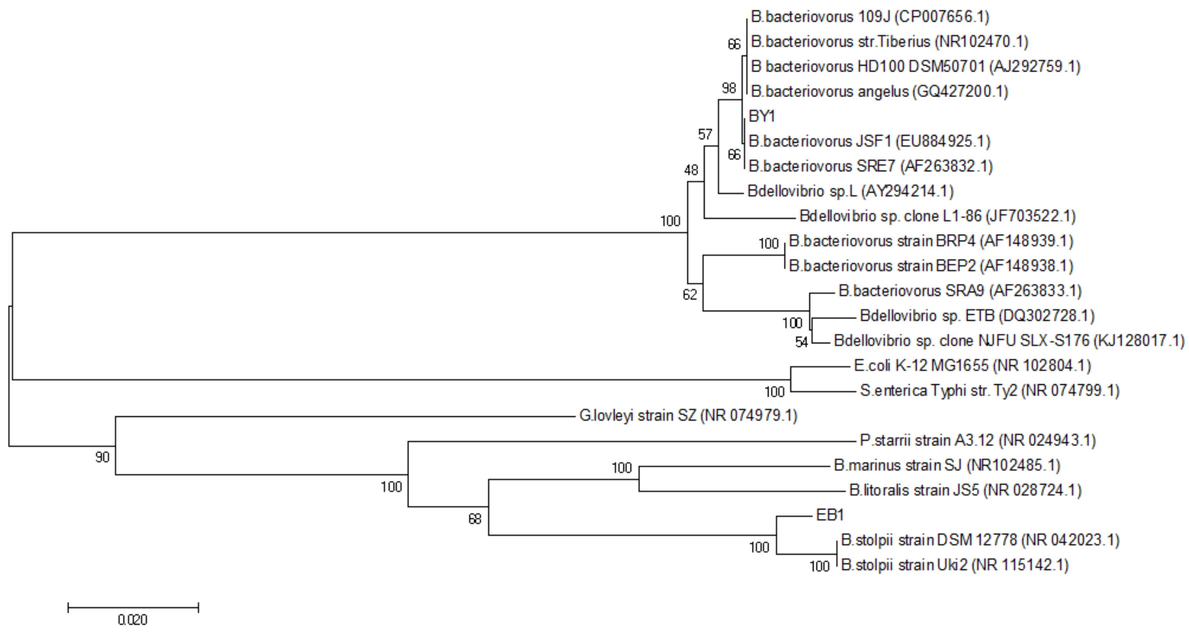

Supplementary Figure 3. Phylogenetic tree showing the classification and taxonomic location of the environmental isolate of predatory EB1 strain. The evolutionary history was inferred using the Neighbor-Joining method. The optimal tree with the sum of branch length = 0.67038433 is shown. The percentage of replicate trees in which the associated taxa clustered together in the bootstrap test (2000 replicates) are shown next to the branches. The tree is drawn to scale, with branch lengths in the same units as those of the evolutionary distances used to infer the phylogenetic tree. The evolutionary distances were computed using the Jukes-Cantor method and are in the units of the number of base substitutions per site. The analysis involved 23 nucleotide sequences. All positions containing gaps and missing data were eliminated. There were a total of 956 positions in the final dataset. Evolutionary analyses were conducted in MEGA7.
